# Shotgun cholanomics of ileal fluid

**DOI:** 10.1016/j.biochi.2012.09.004

**Published:** 2012-09-14

**Authors:** Yingjie Chen, Michael Ogundare, Christopher M. Williams, Yuchen Wang, Yuqin Wang, Gavin W. Sewell, Philip J. Smith, Farooq Z. Rahman, Nuala O’Shea, Anthony W. Segal, William J. Griffiths

**Affiliations:** a Clinical Laboratory, The Second Hospital of Shandong University, Jinan, China; b Institute of Mass Spectrometry, College of Medicine, Swansea University, Singleton Park, Swansea SA2 8PP, UK; c EPSRC National Mass Spectrometry Service Centre, College of Medicine, Swansea University, Singleton Park, Swansea SA2 8PP, UK; d Clinical Laboratory, Jinan Infectious Disease Hospital, Shandong University, Jinan, Shandong, China; e Division of Medicine, University College London, 5 University Street, London WC1 E6JJ, UK

**Keywords:** Bile acid, Lipidomics, Mass spectrometry, Electrospray ionization, Crohn’s disease

## Abstract

In this study we have developed a rapid method for the shotgun analysis of bile acids in intestinal fluid. The method is semi-quantitative, and requires little sample preparation. Bile salts might contribute to the pathogenesis of Crohn’s disease. In a pilot study we demonstrate the method by analysing the bile acid content of ileal fluid from seven Crohn’s disease patients and three healthy controls. The dominant bile acids observed were di and/or trihydroxycholanoates, di- and/or trihydroxycholanoylglycines, di- and/or tri-hydroxycholanoyltaurines, monosulphated dihydroxycholanoates and monosulphated dihydroxycholanoylglycine. The method can be similarly applied to samples derived from other parts of the intestine.

## 1. Introduction

Bile acids represent end products of cholesterol metabolism, they are secreted from the hepatocyte into bile and undergo efficient enterohepatic circulation [1]. The dominating bile acids in human are based on chenodeoxycholic and cholic acid templates and are secreted from the hepatocyte largely conjugated with glycine or taurine [2]. A small fraction of bile acids are not absorbed by the small intestine and may undergo biochemical transformation prior to later absorption from the colon or elimination in faeces.

Bile acids are absorbed in the terminal ileum, a common location of lesions in Crohn’s disease. In this region they are known to increase intestinal permeability [3]. This increase in permeability may facilitate the ingress of antigenic material from the gut lumen into the underlying tissues of the bowel wall, which could then trigger the development of Crohn’s disease lesions in the context of an impaired acute inflammatory response and defective bacterial clearance [4,5]. Mice fed a high fat diet containing cholic acid develop ileocaecal lesions remarkably similar to those observed in Crohn’s disease [6]. The damaging effect of bile acids in Crohn’s disease could be enhanced by abnormalities in membrane lipid composition [7], and changes induced in the intestinal microbiota [8]. No studies have investigated the bile acid composition of ileal fluid in Crohn’s disease.

Here we describe a “shotgun” mass spectrometry method for the rapid profiling of bile acids in ileal fluid. The method is easily adapted for other fluids of the intestine. We demonstrate the method on a small number of patients suffering from Crohn’s disease and healthy controls. The shotgun mass spectrometry (MS) approach utilised here is based on the earlier lipidomic studies of Han and Gross [9] and of Shevchenko and colleagues [10] which utilise direct infusion of sample and electrospray ionisation (ESI) followed by mass and fragment-ion analysis. The advantage of the shotgun approach is its simplicity and ease of use, allowing sample analysis in a matter of minutes providing a high throughput of samples.

## 2. Method

### 2.1. Extraction

Fifty μL of ileal fluid was added to 1 mL of 0.25 M triethylamine sulphate, pH 7 in water containing 10 μL of 23-nor-5β-cholestan-3α,12α-diol (1 mg/mL in methanol) internal standard. The solution was heated to for 60 °C for 5 min, then applied to a previously washed (2 mL methanol/propan-2-ol 1:1, v/v; 2 mL methanol) and conditioned (2 mL 0.25 M triethylamine sulphate, pH 7 in water) SepPak tC_18_ cartridge (100 mg, Waters). The flow-through was discarded. The column was washed with a further 2 mL of water and bile acids eluted with 2 mL of methanol for ESI-MS analysis.

Human samples were collected at UCLH. A 6Fr/220 cm Huibregtse Guiding Catheter (Cook Ireland Ltd) was passed down the forceps channel of the colonoscope and 1 mL of ileal fluid extracted with a syringe and snap frozen in liquid nitrogen. All patients had minimal ileal disease and none had malabsorption or diarrhoea. Ethical approval for this study was from the Joint UCL/UCLH Committees on the Ethics of Human Research (project number 02/0324).

### 2.2. Shotgun ESI-MS

ESI-MS spectra were acquired on an LTQ-Orbitrap XL hybrid linear ion trap – Orbitrap mass spectrometer. ESI was performed using an Advion NanoMate interface utilising silicon-based nano-ESI emitters. Samples were diluted with methanol when signal intensity exceeded the linear dynamic range of the instrument, or to improve the stability of the electrospray. High resolution (100,000, full width at half maximum height), accurate mass (<5 ppm) mass spectra were recorded over an *m/z* range 100–1000. Data was analysed from seven patients with Crohn’s disease and three controls.

## 3. Results

**Fig. 1:**
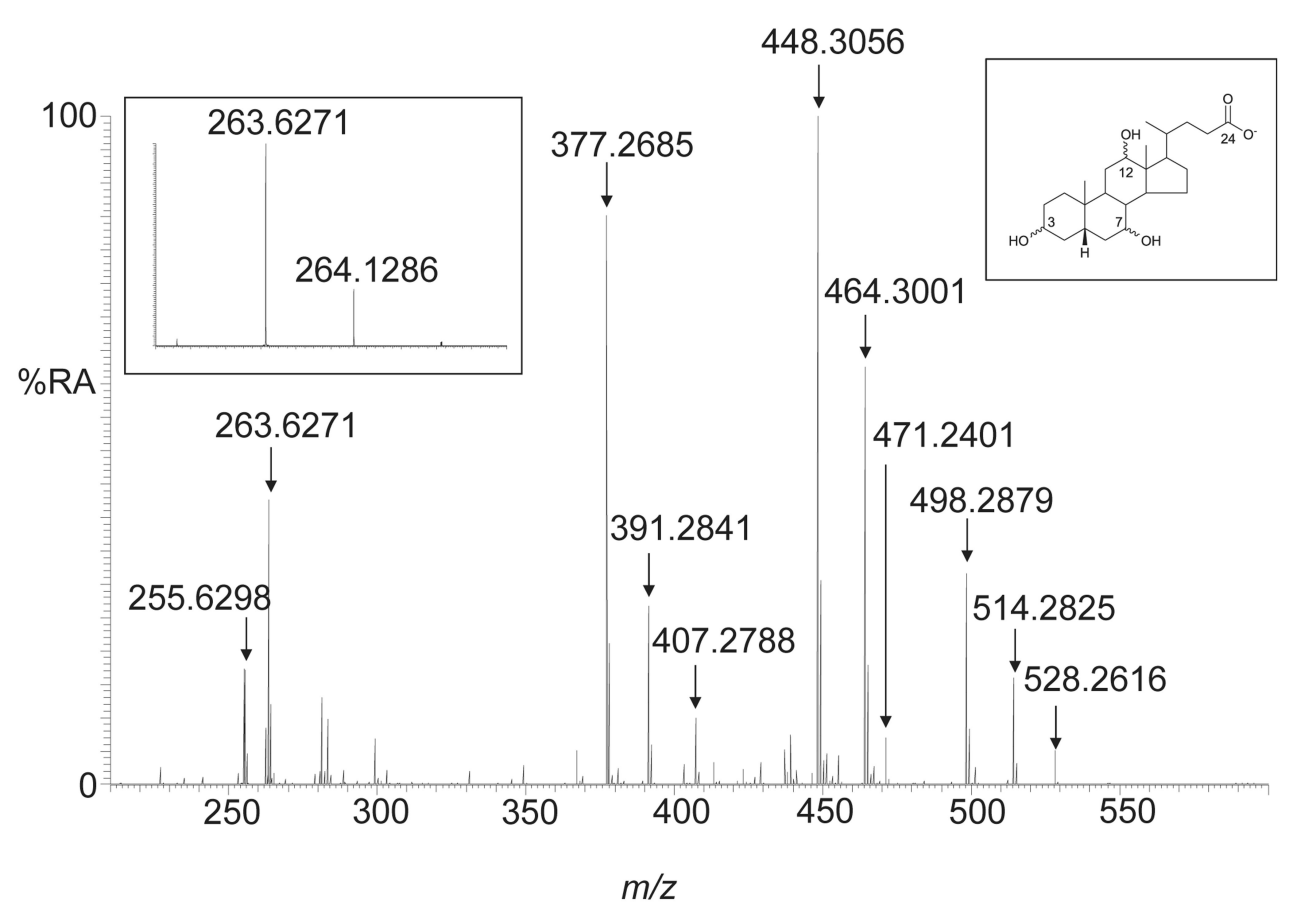
ESI-MS of ileal fluid from a patient suffering from Crohn’s disease. The peaks are annotated as follows: *m/z* 255.6298^2−^, [M – 2H]^2−^ monosulphated mono-hydroxycholanoylglycine (BA-ol-G-S); *m/z* 263.6271^2−^, [M – 2H]^2−^ monosulphated dihydroxycholanoylglycine (BA-diol-G-S); *m/z* 377.2685^−^, [M – H]^−^ 23-nor-5β-cholestan-3α,12α-diol internal standard (IS); *m/z* 391.2841^−^, [M – H]^−^ dihydroxycholanoate (BA-diol); *m/z* 407.2788^−^, [M – H]^−^ trihydroxycholanoate (BA-triol); *m/z* 448.3056^−^, [M – H]^−^ dihydroxycholanoylglycine (BA-diol-G); *m/z* 464.3001^−^, [M – H]^−^ trihydroxycholanoylglycine (BA-triol-G); *m/z* 471.2401^−^, [M – H]^−^ monosulphated dihydroxycholanoate (BA-diol-S); *m/z* 498.2879^−^, [M – H]^−^ dihydroxycholanoyltaurine (BA-diol-T); *m/z* 514.2825^−^, [M – H]^−^ trihydroxycholanoyltaurine (BA-triol-T); and *m/z* 528.2616^−^, [M – H]^−^ monosulphated dihydroxycholanoylglycine (BA-diol-G-S). The inset on the left shows the isotopic pattern of the [M – 2H]^2−^ ion of monosulphated dihydroxycholanoylglycine. The inset on the right shows the trihydroxycholanoate structure. Glycine and taurine conjugation is via an amide bond at C-24, sulphation is usually at C-3. We use the abbreviation system where “BA” corresponds to the cholanoic acid skeleton, if present carbonecarbon double bonds are indicated by “en”, and alcohol substituents by “ol”, “diol”, “triol” etc. Taurine, glycine and sulphuric acid conjugation is indicated by T, G or S, respectively.

Bile acids were annotated based on exact mass (<5 ppm) (Fig. 1). Quantitative estimates were made against 23-nor-5β-cholestan-3α,12α-diol internal standard using response factor determined using a calibration mixture incorporating each class of bile acid present over two orders of concentration.

### 3.1. Crohn’s disease patients

**Fig. 2:**
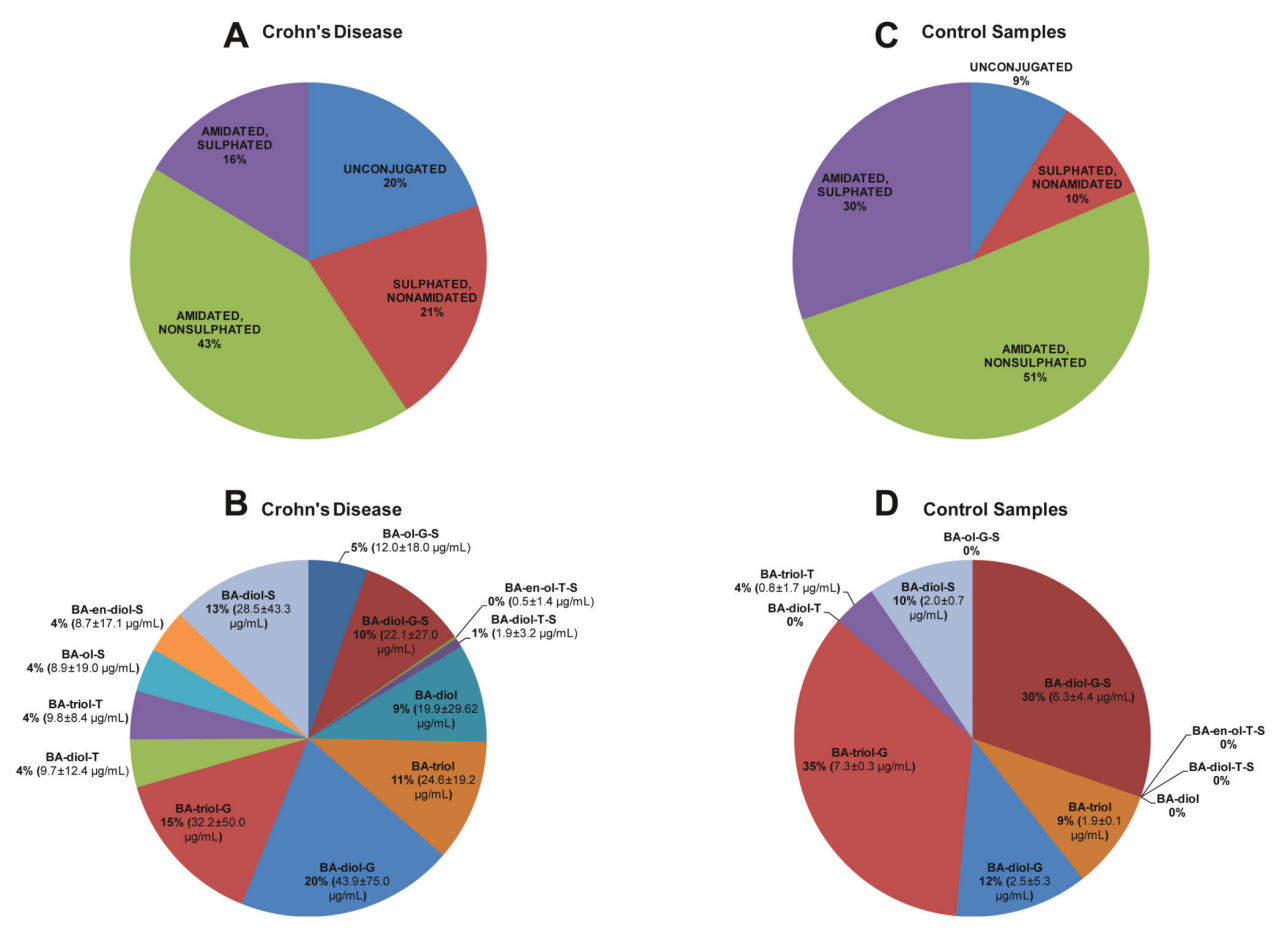
Group and class of bile acids in ileal fluid. Distribution of bile acids according to (A) group and (B) class, from Crohn’s disease patients (*n* = 7). Distribution of bile acids according to (C) group and (D) class, from controls (*n* = 3). Mean concentration ± SD are given.

The level of total bile acids varied considerably across the seven Crohn’s patients studied ranging from 8.9 to 442.8 μg/mL giving a mean value of 222.8 ± 186.9 (mean ± standard deviation, SD) μg/mL. In comparison the coefficient of variation for a quality control sample was less than 15%. A wide variability between subjects is not unexpected as the ileal concentration of bile acids is dependent on a complex set of factors including sampling time after feeding, active absorption of bile acids from the small intestine, and water absorption by the intestine. Bile acids are secreted from the hepatocyte into bile in a predominantly conjugated form as glycine or taurine amidates [1]. In the intestine they undergo complex bacterial biotransformations including deconjugation which occurs in the distal small intestine and they are actively absorbed by the enterocyte of the ileum. In our study we find about 20% (44.5 ± 35.6 μg/mL) of the ileal bile acids in a fully unconjugated form, and a further 21% (46.1 ± 79.3 μg/mL) to be nonamidated but sulphated (Fig. 2A). About 43% (95.6 ± 133.8 μg/mL) of the bile acids in the ileal fluid were amidated but not sulphated, while the final 16% (36.5 ± 37.6 μg/mL) were amidated and sulphated. Shown in Fig. 2B is a breakdown of the individual bile acid classes which go to make up the bile acid groups described above. The major classes are the di- and tri-hydroxycholanoylglycine species (20 and 15% of the total bile acids, respectively), the di- and tri-hydroxycholanoates (9 and 11% respectively) and the monosulphates of dihydroxycholanoate and dihydroxycholanoylglycine (13 and 10% respectively). Only minor amounts of taurine conjugated bile acids were observed.

### 3.2. Control subjects

Only three control subjects were analysed in this study, and the ileal bile acid levels were considerably lower than those of the Crohn’s disease patients (20.8 ± 7.7 μg/mL cf. 222.8 ± 186.9 μg/mL). However, the general pattern of bile acid groups was similar to those of Crohn’s patients (cf. Fig. 2A & C). The class specific patterns were less similar, (cf. Fig. 2B & D) however, with such a low sample number it is unwise to attempt to draw any conclusions from any differences.

## 4. Discussion

In this communication we describe a simple and rapid method for profiling bile acids in intestinal fluids. The method is semi-quantitative and involves a solid phase extraction step followed by high resolution exact mass analysis of the extract. Exact mass measurement (<5 ppm) allows the definition of bile acid class e.g. dihydroxycholanoylglycine, but not the location of hydroxy groups. As it is likely that multiple isomers are responsible for a given bile acid peak in the ESI mass spectrum e.g. the dihydroxycholanoylglycine peak at *m/z* 448.3056 can correspond to a mixture of chenodeoxycholic acid (5β-BA-3α,7α-diol-24-G), ursodeoxycholic acid (5β-BA-3α,7β-diol-24-G) and deoxycholic acid (5β-BA-3α,12α-diol-24-G), only quantitative estimates are made as different isomers do not necessarily give identical response factors. These limitations could be overcome by utilising liquid chromatography-ESI-MS but with the penalty of time and throughput.

## Abbreviations

BA: cholanoic acid skeleton
ESI: electrospray ionisation
MS: mass spectrometer or spectrometry
SD: standard deviation

## References

[1] Hofmann AF (1999). Bile acids: the good, the bad, and the ugly. News Physiol. Sci.

[2] Griffiths WJ, Sjövall J (2010). Bile acids: analysis in biological fluids and tissues. J. Lipid Res.

[3] Fasano A, Budillon G, Guandalini S, Cuomo R, Parrilli G, Cangiotti AM, Morroni M, Rubino A (1990). Bile acids reversible effects on small intestinal permeability. An in vitro study in the rabbit. Dig. Dis. Sci.

[4] Sewell GW, Marks DJ, Segal AW (2009). The immunopathogenesis of Crohn’s disease: a three-stage model. Curr. Opin. Immunol.

[5] Smith AM, Rahman FZ, Hayee B, Graham SJ, Marks DJ, Sewell GW, Palmer CD, Wilde J, Foxwell BM, Gloger IS, Sweeting T, Marsh M, Walker AP, Bloom SL, Segal AW (2009). Disordered macrophage cytokine secretion underlies impaired acute inflammation and bacterial clearance in Crohn’s disease. J. Exp. Med.

[6] Lin JA, Watanabe J, Rozengurt N, Narasimha A, Martin MG, Wang J, Braun J, Langenbach R, Reddy ST (2007). Atherogenic diet causes lethal ileo-cecocolitis in cyclooxygenase-2 deficient mice. Prostaglandin. Other Lipid Mediat.

[7] Sewell GW, Hannun YA, Han X, Koster G, Bielawski J, Goss V, Smith PJ, Rahman FZ, Vega R, Bloom SL, Walker AP, Postle AD, Segal AW (2012). Lipidomic profiling in Crohn’s disease: abnormalities in phosphatidylinositols, with preservation of ceramide, phosphatidylcholine and phosphatidylserine composition. Int. J. Biochem. Cell. Biol.

[8] Chassaing B, Etienne-Mesmin L, Bonnet R, Darfeuille-Michaud A (2012). Bile salts induce long polar fimbriae expression favouring Crohn’s disease-associated adherent-invasive *Escherichia coli* interaction with Peyer’s patches. Environ. Microbiol.

[9] Han X, Gross RW (2005). Shotgun lipidomics: electrospray ionization mass spectrometric analysis and quantitation of cellular lipidomes directly from crude extracts of biological samples. Mass. Spectrom. Rev.

[10] Graessler J, Schwudke D, Schwarz PE, Herzog R, Shevchenko A, Bornstein SR (2009). Top-down lipidomics reveals ether lipid deficiency in blood plasma of hypertensive patients. PLoS One.

